# The impact of different perspectives on the cost-effectiveness of remote patient monitoring for patients with heart failure in different European countries

**DOI:** 10.1007/s10198-024-01690-2

**Published:** 2024-05-03

**Authors:** Hamraz Mokri, Pieter van Baal, Maureen Rutten-van Mölken

**Affiliations:** 1 Erasmus School of Health policy and Management (ESHPM), Erasmus University Rotterdam, Rotterdam, The Netherlands; 2 Institute for Medical Technology Assessment(iMTA), Erasmus University Rotterdam, Rotterdam, The Netherlands

**Keywords:** Economic evaluation, Future costs, Willingness-to-pay, Societal perspective, Healthcare (payer) perspective, Cost estimation, Remote patient monitoring

## Abstract

**Background and objective:**

Heart failure (HF) is a complex clinical syndrome with high mortality and hospitalization rates. Non-invasive remote patient monitoring (RPM) interventions have the potential to prevent disease worsening. However, the long-term cost-effectiveness of RPM remains unclear. This study aimed to assess the cost-effectiveness of RPM in the Netherlands (NL), the United Kingdom (UK), and Germany (DE) highlighting the differences between cost-effectiveness from a societal and healthcare perspective.

**Methods:**

We developed a Markov model with a lifetime horizon to assess the cost-effectiveness of RPM compared with usual care. We included HF-related hospitalization and non-hospitalization costs, intervention costs, other medical costs, informal care costs, and costs of non-medical consumption. A probabilistic sensitivity analysis and scenario analyses were performed.

**Results:**

RPM led to reductions in HF-related hospitalization costs, but total lifetime costs were higher in all three countries compared to usual care. The estimated incremental cost-effectiveness ratios (ICERs), from a societal perspective, were €27,921, €32,263, and €35,258 in NL, UK, and DE respectively. The lower ICER in the Netherlands was mainly explained by lower costs of non-medical consumption and HF-related costs outside of the hospital. ICERs, from a healthcare perspective, were €12,977, €11,432, and €11,546 in NL, the UK, and DE, respectively. The ICER was most sensitive to the effectiveness of RPM and utility values.

**Conclusions:**

This study demonstrates that RPM for HF can be cost-effective from both healthcare and societal perspective. Including costs of living longer, such as informal care and non-medical consumption during life years gained, increased the ICER.

**Supplementary Information:**

The online version contains supplementary material available at 10.1007/s10198-024-01690-2.

## Introduction

Heart Failure (HF) is a complex clinical condition that exhibits the inability of the heart to maintain a healthy blood flow. HF causes both a shorter life expectancy and reduced quality of life. In developed countries, approximately 10% of people above the age of 70 are diagnosed with HF [1]. More than 60% of patients die within five years after the first HF-related hospital admission [2]. Due to the population ageing in western societies, the prevalence of HF is expected to increase further [3]. The Dutch healthcare expenditure on HF in 2017 was estimated to be €817 million, which forms 8% of the total Dutch healthcare expenditure on cardiovascular diseases [4]. In the United Kingdom (UK), the cost of HF is 1–2% of the National Health Service (NHS) budget, of which 60–70% is related to hospitalization [5]. In Germany, the cost of HF accounts for 1.1% of direct health costs [6].

Given the high cost, intensity, and complexity of managing HF and taking the shortage in healthcare staff into account, remote patient monitoring (RPM) interventions are becoming increasingly popular, along with pharmacological treatments. These interventions are defined as services that integrate information communication technology, manifesting either as telemonitoring (the conveyance of physiological data, such as blood pressure, weight, electrocardiographic details, and oxygen saturation, via telephone, digital cable, or wirelessly from the home to healthcare providers) or as routine structured telephone interactions between patients and healthcare providers, with or without the inclusion of physiological data transfer [7]. Their implementation got a boost during the Covid-19 pandemic [8, 9]. Offering RPM to patients with HF may lead to fewer hospital (re)admissions, prevention of death, and a better quality of life, through monitoring of vital signs and early detection of clinical deterioration in patients with HF [10, 11]. Previous studies have evaluated the cost-effectiveness (CE) of various non-invasive RPM technologies for HF [12–23]. They showed that implementing an RPM program is associated with reducing healthcare costs, mainly due to fewer hospital admissions. However, despite the fact that RPM programs are more widely used and have promising results, not much is known about the CE of these interventions for HF condition from a societal perspective. In other words, CE studies of RPM are usually conducted from a healthcare perspective [24].

The healthcare perspective concentrates on costs and benefits directly associated with the healthcare sector. Adopting the societal perspective is deemed essential for making optimal societal decisions. This is particularly crucial, as decisions made from a healthcare perspective may inadequately optimize overall welfare and may not account for resource use outside the healthcare sector [25]. An economic evaluation from a societal perspective is a comparison of the ‘state of the society’ with and without the intervention. This implies that costs within and outside the healthcare sector need to be included, even if they are not related to the disease of interest and occur during years of life gained by the intervention [26]. The inclusion of future, unrelated medical costs has long been debated [27]. Initially, this issue was explored using economic models of representative consumers that aim to maximize lifetime utility taking into account the impact of health on income, which is more closely linked to welfare economics and cost benefit analysis [28, 29]. This has led to differing views on the issue, with some arguing that including costs unrelated to the condition of interest may imply a penalty for interventions that aim to prolong life. However, the prevailing notion is that because future unrelated medical consumption benefits are generally included in the quality-adjusted life year (QALY) gains the associated costs must also be taken into account in order to maintain consistency [30]. Later, models of decision makers facing exogenously determined healthcare budgets also explored this topic [31]. The conclusion from these discussions is that all medical costs in life years gained (not only of the disease at which the intervention is targeted) need to be included in CE analysis from both a healthcare and societal perspective [31]. Including these costs leads to different decisions that result in more health or welfare. Only under strong assumptions (e.g., health spending does not depend on age) can one conclude from such models that so-called future unrelated medical costs can be ignored in cost-effectiveness analysis [29, 32].

The inclusion of future costs of non-medical consumption has been the subject of more recent, renewed debate. Non-medical costs refer to the costs of consumption incurred during the additional years gained due to extended life, such as housing, food, and travel. These costs should be balanced against the productivity gains from the ability to work longer. Although changes in productivity and non-medical consumption in life years gained are relevant from a societal perspective there are concerns about inclusion as the benefits of changes therein are probably not (fully) captured in the QALY [33]. Furthermore, it is unclear to what extent the thresholds used by decision makers take into account these benefits [27, 30, 33]. Countries that adopt a societal perspective in economic evaluations incorporate production gains but overlook the costs of non-medical consumption. This approach is considered inconsistent, as the theoretical arguments for or against including non-medical consumption also apply to production [28, 30]. Moreover, including production gains while excluding non-medical consumption may have distributional consequences, primarily benefiting higher socio-economic groups, which tend to be more productive [28] but also have the highest non-medical consumption throughout their lifecycle. Hence, from a theoretical point of view, it is appropriate to consider the net result of future production minus future consumption. The Health Technology Assessment (HTA) guidelines of different countries include different recommendations with respect to these costs. US HTA guidelines as well as the new Dutch HTA guidelines that became active in January 2024, request the inclusion of future unrelated medical costs. However, the US guidelines are the only ones recommending the inclusion of the future costs of non-medical consumption. Several studies have demonstrated the impact of these costs in economic evaluations in different countries and for different disease areas, such as diabetes mellitus, chronic HF, and chronic kidney disease [28, 34–48]. This impact is especially pronounced when the years of life gained are spent in relatively poor health, which worsens the incremental cost-effectiveness ratio (ICER). Considering that interventions for patients with HF often prolong life and that these additional years of life are spent in relatively poor health, the impact of including these unrelated medical and non-medical costs may be substantial, especially since productivity gains are not to be expected in this high-aged population [27, 28].

In this study, we focus on the impact that the choice of perspective has on the CE of non-invasive RPM interventions for HF. Non-invasive RPM was defined as digital/broadband/satellite/wireless or blue-tooth transmission of physiological and other non‐invasively collected data to the healthcare provider [49]. Given the increased deployment of RPM interventions, it is important for reimbursement authorities and payers to have robust evidence of the costs and effects of this approach from a broad societal perspective. Therefore, this study aims to assess the cost-effectiveness of RPM in managing HF from a societal perspective, compare that to the cost-effectiveness from a healthcare perspective and discuss drivers of the difference between the perspectives. We estimate the CE of RPM compared to usual care (UC) for three countries (the Netherlands, Germany, and the UK) using a health economic model, as these countries differ in the provision and financing of healthcare as well as their guidelines for conducting CE studies.

## Methods

### Patient population

The patient characteristics of this population were based on the patient population of the Trans-European Network-Home-Care Management System (TEN-HMS) study [22]. The TEN-HMS study included 426 patients from twelve main and four satellite hospitals in Germany, the Netherlands, and the UK, who were assigned randomly to receive home telemonitoring, nurse telephone support, or usual care (UC). Patients eligible for inclusion had a recent hospitalization (lasting > 48 h) due to or complicated by worsening HF within the last six weeks, persistent symptoms, left ventricular ejection fraction < 40%, left ventricular end-diastolic dimension > 30 mm/m, and were receiving furosemide ≥ 40 mg/day or equivalent. This study includes detailed information on baseline demographic and social characteristics, clinical history, medication, New York Heart Association (NYHA) functional classification, weight, and physical signs.

### Model structure

**Fig. 1:**
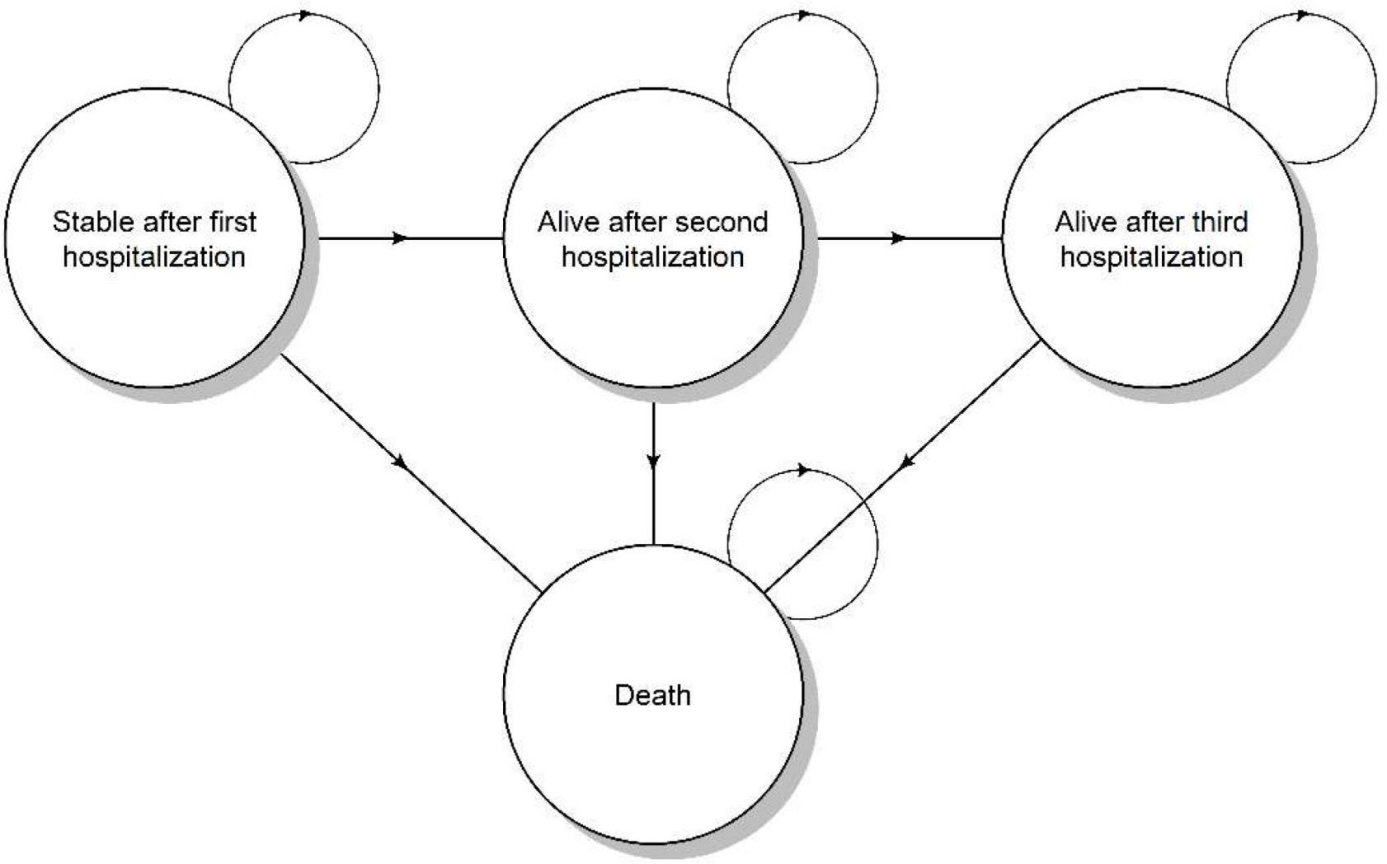
Markov model structure

A cohort-state transition model (Markov model) was developed to estimate the CE of RPM effect for HF compared with UC strategies in three countries, the Netherlands, Germany, and the UK. The model starts with patients that have HF for which they have been hospitalized. The model includes four states, i.e., stable after first hospitalization, alive after second hospitalization, alive after third hospitalization, and death (Fig. 1). We model a cohort of patients with HF with a mean age of 70. The model simulates how the cohort transitions between health states over time due to the occurrence of second and third HF-related hospitalizations and death. The model’s time horizon was lifetime, meaning that patients in the cohort were followed up to age 100, after which the entire cohort was assumed to die in the next cycle. The cycle length was three months. Both the probability of HF-related hospitalization and mortality were age-dependent.

### Treatment arms and transition probabilities

In the UC arm, all-cause mortality rates (e.g., transition probabilities from any state to the death state) were based on a published Weibull distribution that was estimated using patient-level data coming from the TEN-HMS study. Similarly, HF-related hospitalizations rates (e.g., transition probabilities from “stable after first hospitalization” to “alive after second hospitalization” or “alive after second hospitalization” to “alive after third hospitalization”), were derived from a published log-normal distribution that was fitted to the data from the TEN-HMS study [13].

For the intervention arm, risk ratios (RRs) associated with all-cause mortality and HF-related hospitalizations were taken from a Cochrane systematic literature review and meta-analysis of RPM and were applied to the baseline all-cause mortality and HF-related hospitalizations rates [49]. This review by Inglis et al. included 41 studies of either structured telephone support or non-invasive home telemonitoring for people with HF. In this study, we used the non-invasive home telemonitoring results from this review. It was concluded that non-invasive telemonitoring reduced all-cause mortality (RR 0.80, 95% confidence interval (CI) 0.68 to 0.94). The effect of non-invasive telemonitoring on the risk of HF-related hospitalizations was a RR of 0.71 (95% CI 0.60 to 0.83) [49]. Although many studies have shown a positive impact of RPM on all-cause mortality and HF-related hospitalization, the majority of RPM studies have relatively short follow-up periods, typically ranging from 0 to 12 months. The few studies with extended follow-up often reported that the effects did not persist over time [15, 17, 50] due to reduced compliance, non-aligned financial incentives, lack of knowledge about the characteristics of patients suitable for RPM, the type of RPM, and its optimal scheduling. Previous studies of RPM have shown that adherence rates range from 40 to 90%, with adherence decreasing over time [15, 17, 50]. For example, a study by M. Brons et al. reported high patient compliance, starting at 90% and decreasing to 71% after 12 months [51]. Therefore, in the base-case analysis, we assumed that the effect of RPM on all-cause mortality and HF-related hospitalization is waning over time. In the first year, we used the effects found in the study by Inglis et al. [49]. Then, we reduced it to 70% in the second year and 30% in the third year, assuming no effect from the fourth year onwards.

### Utilities

The treatment arm-independent utility values were derived from the EuroQol 5 Dimension (EQ-5D-3L) data in the TEN-HMS database [12, 22]. The weighted average utility value was 0.6970, which was applied to patients in the “stable after first hospitalization” state. Utility decrements were assigned for the hospitalization states. For the ‘alive after second hospitalization” and “alive after third hospitalization” states, we applied permanent utility decrements estimated by Kansal et al. [52, 53], who concluded that disutilities for patients with one or two hospitalizations were similar. The utilities are shown in Table 1.

### Resource use and costs

The current study compares the CE of RPM from a societal perspective with that from a healthcare perspective. The societal perspective incorporates four cost categories: (1) intervention costs and HF-related hospitalization and non-hospitalization costs (HF-related medical costs), (2) medical costs due to other diseases (current and future unrelated medical costs), (3) informal care costs, and (4) costs of non-medical consumption (current and future unrelated non-medical costs). The healthcare perspective incorporates cost categories one and two. All cost inputs were specified per country, inflated to 2020 price levels [54], and discounted by country-specific discounting rates. All annual costs were converted to a 3-monthly cycle to be used in the model, and hospitalization costs were assigned per event. Furthermore, except for intervention costs, all other costs were assumed to be treatment arm-independent [55, 56]. . Intervention costs were assigned to patients in the “stable after first hospitalization” state in the treatment arm and only for the first three years. For the base-case, the costs of RPM, including the costs for setup, equipment, and service fees, were determined based on an economic evaluation of RPM conducted by Grustam et al. [12] (Table 1). As treatment effectiveness declines due to decreased patient adherence, intervention costs also decrease, with fixed costs such as technical infrastructure remaining constant over three years and variable costs, including personnel and telehealth nursing costs, diminishing with reduced patient device usage (i.e., 100%, 70%, 30%, and 0 from the fourth year onwards). In two previous RPM studies, variable costs constitute a substantial portion of the intervention costs (60–78%). To be conservative, we assumed the proportion of variable costs to be 60%.

HF-related hospitalization and non-hospitalization costs were obtained from different sources presented in Table 1. HF-related hospitalization costs were defined as costs associated with hospital admission due to HF. Second and third hospitalization costs were assumed to be the same. HF-related non-hospitalization costs included maintenance treatment costs (physician visits, medication, and cardiologist visits). In addition, because our study focuses on a patient population that is on average approximately 70 years old and generally not actively employed, we did not consider productivity costs in our analysis.

Other medical costs unrelated to HF were defined as costs due to other diseases than HF (such as the treatment of dementia). These costs also include the costs of hospital admissions for other reasons than HF. For the Netherlands and the UK, these other medical costs were obtained from the Dutch Practical Application to Include Disease cost (PAID) tool version 3 and the UK PAID Tool version 1, respectively [57, 58]. These tools estimate the additional costs as a result of a longer lifespan after treatment, taking into account that healthcare spending is highest in the last year of life. For Germany, annual healthcare spending per capita by sex, age, and last year of life was used to estimate these costs [58]. To avoid double counting, the costs of HF were subtracted from these sources. Informal care costs were based on a study by Santi et al. [59]. They utilize the Survey of Health, Ageing and Retirement in Europe (SHARE) data to predict the probability of using informal care and the daily number of hours of informal care use [60], depending on age, sex and proximity to death. The latter is an important predictor because the weekly use of informal care and daily hours of informal care were found to increase when approaching death [57, 61]. The costs of informal care use for the different European regions were then calculated subsequently by combining the probability of using informal care weekly and the daily number of hours of informal care use. The unit cost for informal care, which is based on a standard rate of formal caregivers providing similar activities as informal caregivers, is equal to €22.8 per hour in the UK, €13 per hour in Germany and €13.5 per hour in the Netherlands [59]. Both estimates of informal care and unrelated medical care costs were linked to survival predictions in different scenarios. Note that since both unrelated medical costs and costs of informal care are highest in the last phase of life, the postponement of death also results in the postponement of some of these costs.

Age-specific costs of non-medical consumption (e.g., costs of housing, clothing, food) for the Netherlands were driven by the Dutch PAID tool. Costs of non-medical consumption for Germany and the UK were estimated from national household consumption surveys in each country [36, 57, 58]. All age-dependent and country-specific input parameters (e.g., informal care costs, non-medical costs, and HF-unrelated medical costs) for the base-case analysis are presented in the Appendix.

### Model outcomes

We adhered to the Consolidated Health Economic Evaluation Reporting Standards 2022 (CHEERS 2022) guidelines for reporting of CE analysis [62]. We calculate the following outcomes in our model: total cost, total life years, total quality-adjusted life years (QALYs), and the incremental cost-effectiveness ratio (ICER). The results are presented from a healthcare perspective with and without other medical costs and a societal perspective with and without non-medical consumption. The ICER was determined by the difference in costs between RPM and UC, divided by the difference in QALYs, and compared to various willingness-to-pay thresholds ranging from €20,000 to €50,000 per QALY. The threshold value against which the cost effectiveness of the intervention is judged depends on the perspective that is adopted [63]. Thresholds for the societal perspective are usually derived from willingness to pay studies reflecting the consumption value of health (demand-side threshold). Thresholds for a healthcare perspective are nowadays often derived from estimates of the marginal productivity of the (health)care system) indicating potential health losses due to displacement of healthcare if new interventions need to be financed from a fixed healthcare budget (supply-side threshold).

Previous studies have made an attempt to estimate a country-specific cost-effective threshold for both demand-side and supply-side thresholds [64–66]. These studies suggest that cost-effectiveness thresholds from a societal perspective are generally found to be higher than from a healthcare perspective.

For the Netherlands, the Dutch National Healthcare Institute (ZIN) uses a threshold for a societal perspective that increases as the severity of disease (proportional shortfall) increases [67]. For HF, this would correspond to a threshold value of €50,000 per QALY gained. For the UK, the National Institute for Health and Care Excellence (NICE) uses an estimated threshold of between £20,000 and £30,000, which is applicable when using a healthcare perspective [68]. NICE’s severity modifier gives additional weight to health benefits in the most severe conditions. It is based on the absolute and proportional QALY shortfall, whichever is the highest. Absolute QALY shortfall refers to the future health lost by individuals with the condition compared to the expected future health without the condition. It is calculated by subtracting the total QALYs expected with current treatment from the general population’s expected QALYs. Proportional QALY shortfall represents the proportion of future health lost by individuals with the condition [69].For Germany, the Institute for Quality and Efficiency in Health Care (IQWiG) does not apply any particular/specific threshold value for the ICER.

For the Netherlands, we also used a healthcare perspective threshold (supply-side threshold), which was estimated to be €41,000 per QALY gained in the context of cardiovascular disease [70]. For the UK and Germany, thresholds indicating the marginal productivity of health care which are relevant for a healthcare perspective were taken from a study by Woods et al. [64]. The country-specific thresholds were converted to 2020 euros and are equal to €19,675, and €24,167 for the UK and Germany, respectively. The guidelines in the UK and Germany do not recommend the societal perspective, and corresponding cost-effectiveness thresholds are not provided in the country-specific guidelines. Therefore, we used estimates derived by Riviere et al. [71] and converted them to 2020, which resulted in an estimate of €33,644 for the UK, and €41,774 for Germany, respectively. These estimates were based on the rate of increase in health spending per capita and life expectancy at the population level resulting from the adoption of new interventions with specific ICERs. At a very high policy level, the development of the rate of increase in health spending and life expectancy in a particular country indicates a collective willingness of society to pay for the increase in the population’s life expectancy.

### Sensitivity and scenario analysis

We performed one-way sensitivity analyses, in which we estimated the ICER’s sensitivity to changes in input parameters related to transition probabilities, costs, the utility of the “alive after the first hospitalization” state, and treatment effects. We used a 20% increase (upper bound) and a 20% decrease (lower bound) for the model-input parameters on costs and utility. The uncertainty around the treatment effects (i.e., RR) for all-cause mortality and HF-related hospitalization were derived from the lower and upper bounds of the 95% CI as estimated in the study by Inglis et al. [49]. Probabilistic sensitivity analysis (PSA) was performed by running 1000 Monte-Carlo simulations. In each simulation, a value for each parameter was randomly drawn from the probability distribution of the model parameters (costs, treatment effect, transition probabilities, and utilities). The parameters of the distributions used in the PSA are shown in Table 1. We plotted the results in a CE plane.

Since the Cochrane review of the evidence on the effects of RPM is only based on short-term studies in which 38% of the studies had a follow-up time of less than six months [49], we have implemented different scenarios regarding the duration of the treatment effect. In the first scenario analysis, we assumed that patients receive RPM for one year, after which there were no intervention costs but also no more effects. In the second scenario analysis, we assumed that RPM would be continued every year, which means that RPM will continuously influence all-cause mortality and HF-related hospitalization (i.e., the treatment effect is applied every year). In this scenario, the intervention costs were allocated continuously for the lifetime. Furthermore, in the third scenario analysis we repeated the base-case analysis with higher intervention costs based on a study by Thokola et al. [14], which was included for three years (i.e., 100%, 70%, 30%, and 0 from the fourth year onwards). They report RPM costs of £1,288 per patient per 6 months, which were obtained from an expert advisory group [14]. We have adjusted these costs via the consumer price index from 2011 to 2020 and converted it to euros, resulting in intervention costs of €823 per patient per 3 months.

**Table 1:** Input parameters in base-case analysis

Variable group	Variable description	Mean value	Probability sensitivity analysis SE Distribution		Source
Model settings	Discount rates (costs, %) - The Netherlands - Germany - The UK Discount rates (effects, %) - The Netherlands - Germany - The UK	4 3 3.5 1.5 3 3.5	N/A N/A	Fixed Fixed	[55, 67] [55, 67]
Treatment effect	All-cause mortality HF-related hospitalization Risk ratio all-cause mortality Risk ratio HF-related hospitalization	Weibull Log-normal 0.80 0.71	Covariance matrix* Covariance matrix (95% CI 0.68 to 0.94) (95% CI 0.60 to 0.83)	Multivariate normal Multivariate normal Log-normal Log-normal	[13] [13] [49] [49]
Utility	Utility for “stable after first hospitalization” state Utility decrements second hospitalization Utility decrements third hospitalization	0.6970 0.076 0.074	0.006 0.007 0.013	Beta Beta Beta	[12] [52, 53] [52, 53]
Costs (€)	Intervention costs per 3 months per patient	417	20% of the mean	Gamma	[12]
	High intervention costs scenario	823	-	-	[14]
	HF-related hospitalization (per admission) - The Netherlands - Germany - The UK	4,937 4,635 2,511	20% of the mean	Gamma	[13] [16] [16]
	HF-related non-hospitalization costs per 3 months - The Netherlands - Germany - The UK	378 393 565	20% of the mean	Gamma	[44, 72] [73] [72]
	Informal care unit costs - The Netherlands - Germany - The UK	13.5 13 22.9	20% of the mean	Gamma	[59, 74]
	Probability of using informal care	Age, gender, and TTD* specific	Covariance matrix	Multivariate normal	[59, 74]
	Daily number of hours of informal care use	Age, gender, and TTD specific	Covariance matrix	Multivariate normal	[59, 74]
	Costs of non-medical consumption	Age-specific	3% of the mean**	Normal distribution	[36, 57, 58]
	Other medical costs	Age- and gender-specific	20% of the mean	Normal distribution	[36, 57, 58]

N/A: not applicable, HF: heart failure. All costs are reported in euros and adjusted to 2020 price levels. TTD: time-to-death (proximity to death). (*) The covariance matrix refers to the correlation between the hyper parameter of the mentioned distributions. These hyper parameters where jointly modelled using a multivariate normal distribution. (**) The 95% prediction intervals and standard deviation were driven by a study by K. Kellerborg et al. [75]

## Results

### Base-case analysis

**Fig. 2:**
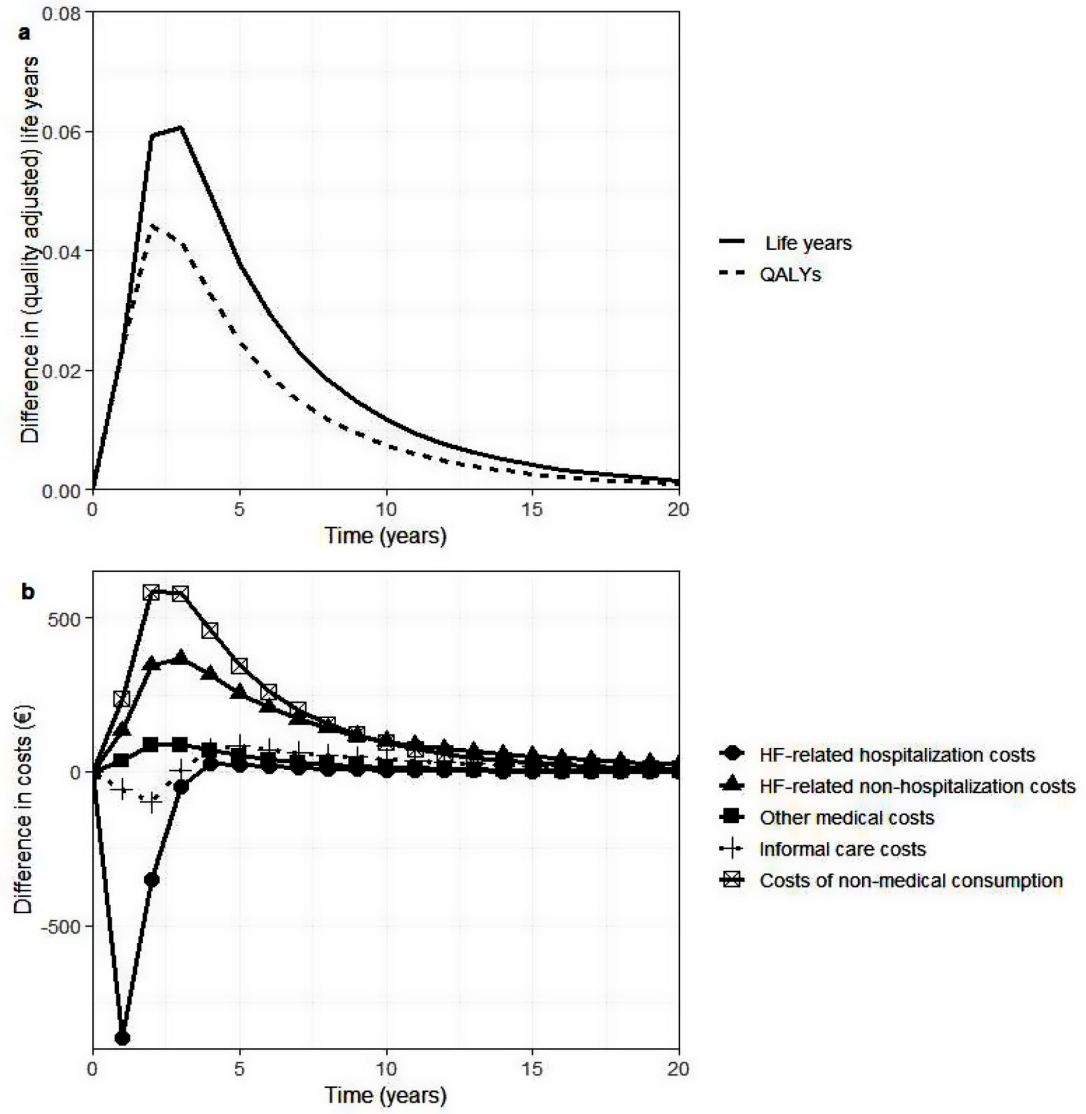
Difference in QALYs, life years and costs per patient between UC and RPM in the Netherlands over time. QALYs, Quality-adjusted life years. (a) difference in QALYs and life years per patient between UC and RPM in the Netherland, (b) difference in cost per patient between UC and RPM in the Netherlands

Figure 2 shows the difference in discounted QALYs, life years, and costs per patient between UC and RPM in the Netherlands over time. As the intervention affects all-cause mortality in the first and second year, QALYs and life years gained are highest in the first years after intervention. As the effectiveness of the intervention wanes, the difference between the treatment arms becomes smaller. In the first years, there are savings in HF-related hospitalization costs and informal care costs. Because informal care costs are modelled as a function of time-to-death, these savings in informal care costs result from fewer deaths in the RPM group. This lower mortality also explains the higher costs of non-medical consumption in these first years. The peak in the total costs in the first year is due to the intervention costs, which were included in the first year.

Lifetime CE results of the base-case analysis are presented in Table 2. RPM results in a discounted life expectancy gain of 0.38 years for the Netherlands, 0.34 years for the UK and 0.35 for Germany. The gain in discounted QALYs from RPM is 0.26 for the Netherlands, 0.24 for the UK and Germany. The difference in life expectancy and QALYs between countries is due to the different country-specific discounting rates. Table 2 shows that using RPM leads to savings in hospitalization costs resulting from the reduction in hospital admissions. In all countries, however, these savings were offset by the increase in the other cost categories due to the increase in life expectancy. In the UK, the increase in HF-related non-hospitalization costs and informal care costs was greater than in the other two countries because of higher unit costs. The increase in costs of non-medical consumption was greater in Germany than in the other two countries because the unit costs of non-medical consumption by age were higher in Germany.

Lifetime incremental costs (including the intervention costs) were a factor 2 to 3 higher from a societal perspective than a healthcare perspective, mainly driven by the costs of non-medical consumption, followed by the other medical costs. Excluding the costs of non-medical consumption led to a greater reduction in total costs in Germany and the UK because these costs are higher in these two countries than in the Netherlands.

When comparing the ICERs of RPM of €12,977, €11,432, and €11,546 per QALY from a healthcare perspective in the Netherlands, the UK, and Germany, respectively, to a willingness-to-pay (WTP) threshold of €20,000 per QALY, RPM was found to be cost-effective. From a societal perspective, including costs of non-medical consumption, ICERs rise to €27,921, €32,263, and €35,258 per QALY in the Netherlands, the UK, and Germany, respectively. This means that RPM would be cost-effective from a societal perspective in the Netherlands, which uses a threshold value that increases as the severity of disease increases, leading to an applicable threshold of €50,000 per QALY. Assuming the WTP estimations from the study by Riviere et al., amounting to €33,644 for the UK and €41,774 for Germany, reflect the cost-effectiveness considerations from a societal perspective in these countries, it implies that RPM is deemed cost-effective. Using NICE’s severity modifier to calculate the absolute QALY shortfall and the proportional QALY shortfall did not change our cost-effectiveness results. The QALY weight, in our case, is equal to one [69, 76].

**Table 2:** Lifetime discounted cost-effectiveness results of RPM vs. UC for the base-case analysis

	The Netherlands		The UK		Germany	
	UC	Intervention outcome incremental to UC	UC	Intervention outcome incremental to UC	UC	Intervention outcome incremental to UC
Life years (LY)	3.43	0.38	3.18	0.34	3.24	0.35
QALYs	2.18	0.26	2.03	0.24	2.06	0.24
HF-related hospitalization costs	€ 5,134	-€ 1,143	€ 2,639	-€ 581	€ 4,926	-€ 1,074
HF-related non-hospitalization costs	€ 4,733	€ 507	€ 7,196	€ 776	€ 5,093	€ 553
Other medical costs	€ 23,418	€ 2,760	€ 11,180	€ 1,259	€ 17,238	€ 2,060
Informal care costs	€ 6,364	€ 519	€ 10,748	€ 878	€ 6,128	€ 500
Costs of non-medical consumption	€ 31,640	€ 3,384	€ 38,693	€ 4,083	€ 48,495	€ 5,274
Total intervention costs	-	€ 1,265	-	€ 1,269	-	€ 1,272
Total costs from the societal perspective including costs of non-medical consumption	€ 71,288	€ 7,293	€ 70,456	€ 7,684	€ 81,881	€ 8,586
ICER from societal perspective including costs of non-medical consumption	-	€ 27,921	-	€ 32,263	-	€ 35,258
ICER from societal perspective excluding costs of non-medical consumption	-	€ 15,031	-	€ 15,004	-	€ 13,796
ICER from healthcare perspective	-	€ 12,977	-	€ 11,432	-	€ 11,546
ICER from healthcare perspective, excluding other medical costs	-	€ 2,419	-	€ 6,100	-	€ 3,129

**Fig. 3:**
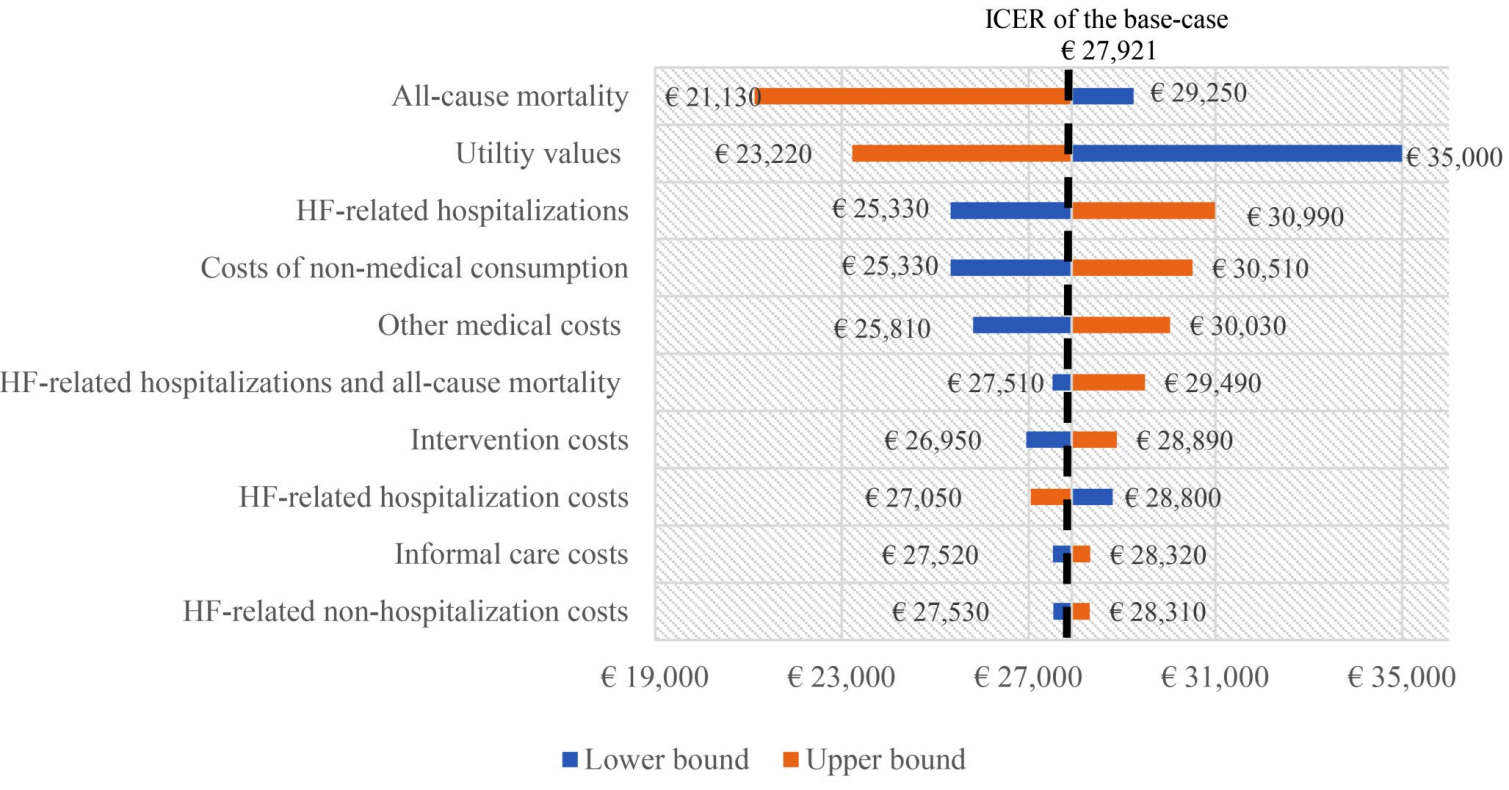
Tornado diagram for the one-way sensitivity analysis showing the variation in the base-case cost-effectiveness results for the Netherlands and from a societal perspective. RR, risk ratio. A larger bar indicates a greater impact on the ICER. The dotted line indicates the base-case ICER

Figure 3 shows the results of the one-way sensitivity analysis of the Netherlands; the Tornado diagrams of the other two countries are shown in the Appendix and show similar results. The variation of all-cause mortality rate, utility values, and HF-related hospitalizations rate had the biggest impact on the ICER. As the treatment effect on HF-related hospitalizations decreases (e.g., RR close to one, upper bound), the ICER increases. When the utility values of the health state ‘alive after first hospitalization’ decreased by 20%, the ICER increases. The ICER is more sensitive to changes in the costs of non-medical consumption and other medical costs than to changes in other types of costs.

**Fig. 4:**
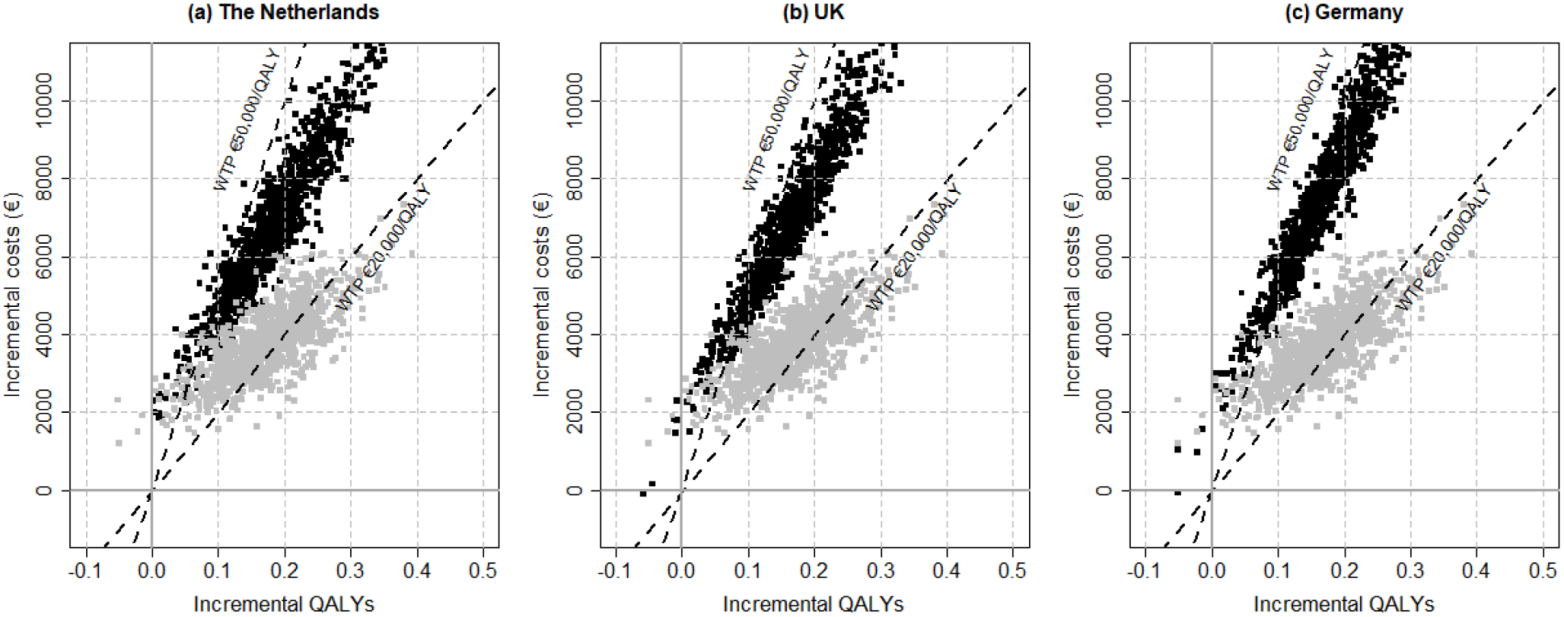
Cost-effectiveness plane for RPM versus UC in the Netherlands, the UK, and Germany, from a societal (black) and a healthcare perspective (grey) The lower dashed line indicates a WTP of €20,000 per QALY and the upper dashed line indicates a WTP threshold of €50,000 per QALY. WTP, willingness to pay

**Fig. 5:**
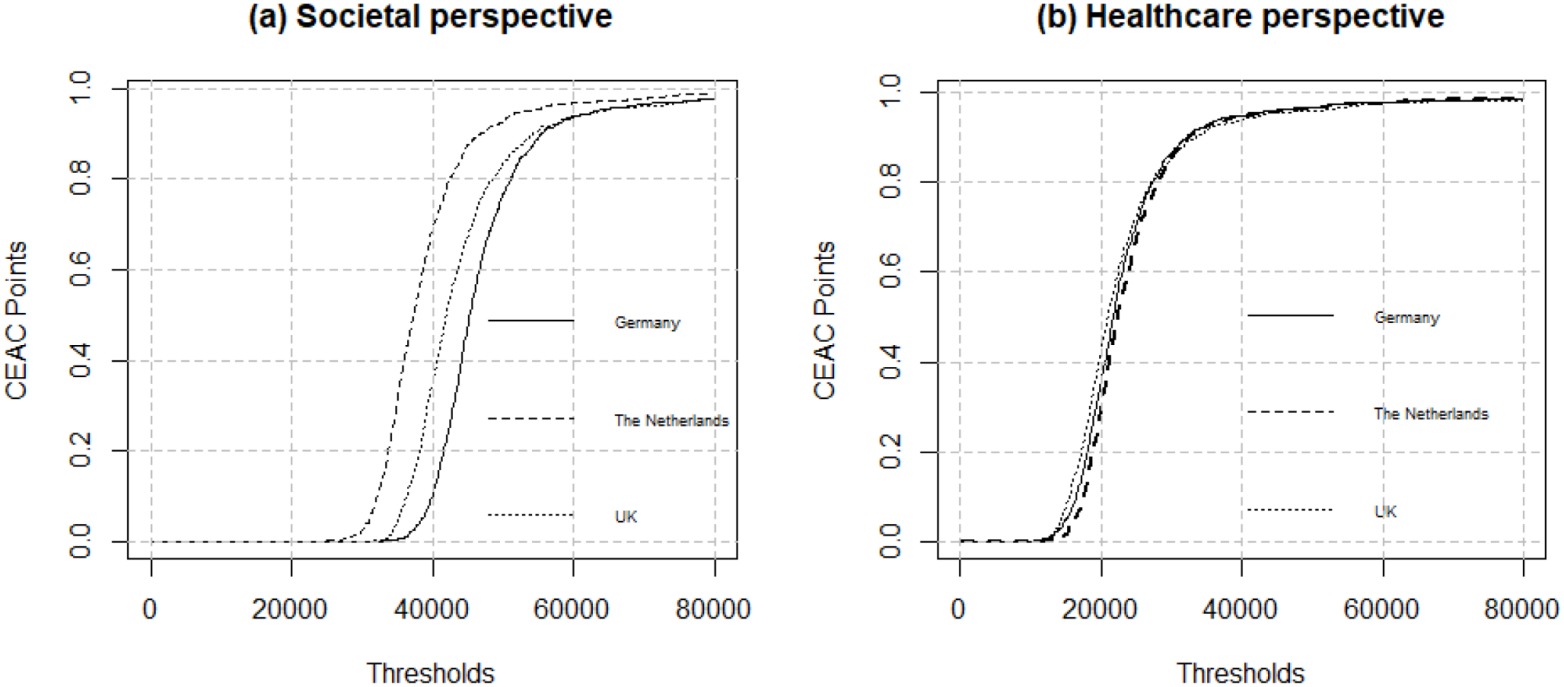
Cost-effectiveness acceptability curve (CEAC) for RPM versus UC in the Netherlands, the UK, and Germany from a societal and a healthcare perspective. WTP, willingness to pay

Figure 4 shows the results of the PSA for all countries on the CE plane. The PSA shows a cluster of results in the northeast quadrant. Based on Fig. 5, the probability of RPM being cost-effective at the €50,000 WTP threshold from a societal perspective in the Netherlands, UK, and Germany is 93%, 83%, 77%, respectively. The probability of RPM being cost-effective from a healthcare perspective at the €20,000 WTP threshold in the Netherlands, UK, and Germany is 29%, 42%, 35%, respectively.

Table 3 shows the results of scenario analyses per country. In the first scenario, when it was assumed that RPM is only effective for one year, the ICER decreased slightly in all countries compared to the base-case. In the second scenario, assuming a lifelong continuation of RPM led to the highest increase in discounted QALYs between all scenarios (e.g., ranging from 0.54 in the UK to 0.64 in the Netherlands). However, in this scenario, the gains in QALYs were offset by a much larger cost difference between the RPM and UC arms due to longer life expectancy, resulting in higher ICERs than in the baseline scenario. In the third scenario, repeating the base-case scenario with higher intervention costs (from a baseline value of €417 to €823 per patient per 3 month), led to the highest ICERs of all scenarios.

**Table 3:** Discounted cost-effectiveness results of RPM vs. UC for all scenarios

	The Netherlands	The UK	Germany
**Base-case**			
- Difference in QALYs	0.26	0.24	0.24
- Difference in life years	0.38	0.34	0.35
- Difference in costs	€7,293	€7,683	€8,586
- ICER	€27,921	€32,263	€35,258
**Intervention is implemented for one year**			
- Difference in QALYs	0.16	0.15	0.15
- Difference in life years	0.23	0.21	0.21
- Difference in costs	€4,350	€4,765	€5,138
- ICER	€26,514	€31,474	€33,289
Lifelong continuation of the intervention*			
- Difference in QALYs	0.64	0.54	0.56
- Difference in life years	0.97	0.83	0.86
- Difference in costs	€19,255	€17,290	€22,137
- ICER	€ 30,103	€31,687	€39,053
**High intervention costs scenario**			
- Difference in QALYs	0.26	0.24	0.24
- Difference in life years	0.38	0.34	0.35
- Difference in costs	€8,522	€8,916	€9,822
- ICER	€32,627	€37,439	€40,335

ICER, Incremental cost-effectiveness ratio, QALYs, quality-adjusted life years. Costs are discounted by country-specific discounting rates and inflated to 2020 price levels. (*) In this scenario the intervention costs were also included for a lifetime

## Discussion

This study investigated the cost-effectiveness of RPM in the management of HF in the Netherlands, the UK, and Germany from both a healthcare and a societal perspective. Our results suggest that there is potential for RPM to be cost-effective in the management of HF from both a healthcare and a societal perspective in all three countries. It also shows the impact of including costs of living longer, such as the unrelated medical costs, costs of informal care, and costs of non-medical consumption during the life years gained in a cost-effectiveness analysis. Including the latter cost category leads to the greatest increase in the ICER. Since the threshold value is often higher from a societal perspective, this does not necessarily change an adoption decision.

From a societal perspective, all costs, and benefits, regardless of who incurs them are considered, in our study, this implied that costs of informal care and non-medical consumption were included. Especially the latter cost category had a large impact on the ICER. The inclusion of these costs in a cost-effectiveness analysis is controversial. However, from a theoretical point of view, the costs of non-medical consumption are relevant from a societal perspective. Excluding these costs generates a bias toward favouring life-prolonging interventions over interventions that increase quality of life [27, 28]. One could argue that when we include the costs of non-medical consumption, we should also include the benefits of non-medical consumption. These benefits might implicitly be captured by the EQ-5D, but it is unclear to which extent, as the EQ-5D was developed to measure and value the health-related quality of life and not quality of life in general and there is currently no strong (empirical) evidence on the extent to which the EQ-5D can effectively capture benefits from non-medical consumption. The incorporation of future unrelated medical costs may potentially disadvantage life-prolonging interventions for the elderly and individuals already in poor health or with elevated healthcare expenses. While recognizing the significant impact of including future unrelated medical costs, we argue that disregarding real costs is not an appropriate response to such concerns but rather a necessary input for an ethical debate. Moreover, emphasizing the inclusion of future unrelated medical costs in cost-effectiveness analyses is essential, as neglecting these costs may lead to biased comparisons between life-prolonging interventions and those focusing solely on improving quality of life, potentially resulting in sub-optimal resource allocation and diminished overall health and welfare. The impact of including informal care costs on the results was less strong, but our findings show that these costs do matter and are similar in size to the costs of treating patients with HF outside of the hospital. Here, it should be noted that the costs of informal care were based on SHARE data from the general population and not patients with HF, so these costs might be underestimated.

In addition to different theoretical underpinnings [77, 78], thresholds from both a societal and healthcare perspectives have been proven difficult to estimate empirically [63, 64, 79–81]. These empirical difficulties have resulted in a wide range of threshold estimates from both the societal and healthcare perspective. In our study, we have been pragmatic and have used available country specific threshold estimates that seemed most relevant for our case study. Hence, the estimated ICERs in our study have been compared with two conceptually different thresholds. In the Netherlands, for example, where the applicable WTP was €50,000 per QALY (based on a societal perspective), RPM was found to be cost-effective. In the UK, where the applicable WTP was £20,000-£30,000 (based on a healthcare perspective), the ICER was slightly lower than the upper limit of the threshold value. (i.e., €32,263 or £28,703 based on 2020 exchange rate), which means that RPM is cost-effective at this applicable WTP.

While medical practice for HF is quite similar in the Netherlands, the UK, and Germany [82, 83], the total costs associated with HF are different. This difference may be due to differences in the organization of the healthcare system (e.g., the type or amount of formal care) and the healthcare funding, pricing, and reimbursement system. For example, while costs of HF-related hospitalization are higher in the Netherlands and Germany than in the UK, HF-related non-hospitalization costs - mainly consisting of outpatient visits - are higher in the UK. The costs of non-medical consumption were higher in Germany and the UK, which resulted in a higher ICER for these countries from a societal perspective. However, when we took a healthcare perspective, Germany had slightly lower ICER than the Netherlands, which was due to higher other medical costs in the Netherlands. A potential reason for these higher other medical costs might be that in the Netherlands, unlike in the UK and Germany, some long-term care costs, such as the costs of nursing homes, are included in the other medical costs. In the UK, a much higher proportion of long-term care expenditure comes from private sources, which can result in higher costs of non-medical consumption. Furthermore, the UK appears to have much higher rates of informal care than the Netherlands and Germany [84], which in our analysis, resulted in higher informal care costs when we took a societal perspective.

Previous studies investigated the CE of different RPM interventions in the Netherlands, such as (home) telemonitoring and telephone support by nurses [7, 12, 21, 49, 85–87]. The estimated ICER range from €12,479 to €40,321. Nevertheless, conducting direct comparisons of cost-effectiveness results presents a challenge due to variations in time horizon, patient demographics, disease severity, perspective, and RPM service protocols. With study durations typically under one year, the long-term cost-effectiveness of RPM remains uncertain. Therefore, the CE of these types of interventions is sensitive to the assumptions made about their long-term effects. Therefore, the CE of these types of interventions is sensitive to the assumptions made about their long-term effects. In this study, we performed various scenario analyses on the duration of RPM effectiveness. When RPM was assumed to be continuously effective, because of much higher intervention costs, the ICER was higher than the base-case. This was due to a much larger difference in the costs between RPM and UC in the former scenario.

As with any modelling study, our analysis has limitations determined by data availability and associated assumptions. The first limitation of this study is the combination of utilities for a stable HF state and utility decrements for HF hospitalization states from different sources. The TEN-HMS study did not explicitly provide estimates on utility decrements during HF hospitalizations. To address this gap, we rely on a study by A.R. Kansal et al. [52, 53], which reports decrements for one, two, or three or more HF hospitalizations. These were the decrements that we used, and we indeed make the assumption that these can be applied to the utilities of the TEN-HMS study, which seems reasonable given that the baseline utilities in TEN-HMS and Kansal et al. for the patient population in our study were comparable (0.687 vs. 0.666). The second limitation of our study is that the country-specific sources we used to obtain cost inputs were diverse. Cost categories are not defined in exactly the same way across countries and may include different types of resource use. Countries also differ in UC management in terms of content and the number of visits to cardiologists and general practitioners, and medications. This is most apparent for the informal care costs. HF medications and their reimbursement regulations may also vary by country. However, these differences do not affect the overall conclusion since we calculate total costs. In addition, since we had no data on the impact of RPM on quality of life, it was assumed that utilities in our study were only dependent on health state but independent of the treatment group. Hence, differences in QALYs between the groups result from differences in the distribution of patients across health states. If being monitored at home improves the quality of life in general, the QALY gains of RPM might be underestimated. Finally, RPM is increasingly positioned as a labour-saving digital technology that supports self-management and substitutes routinely scheduled visits to cardiologists and specialized nurses. However, there is little evidence in the literature to substantiate this assumption. A systematic review by Auener et al. shows that RPM leads to an increase in non-emergency outpatient department visits and does not significantly impact the number of emergency visits [88]. Therefore, this study, we assumed the same HF-related non-hospitalization costs (including outpatient visits) for both treatment arms. However, as the relative costs of an outpatient visit are low, we expect a small impact of this assumption on the ICER. In current HF care, RPM seems to be positioned as an add-on to standard care, but some more recent forms of RPM, which include diagnostic algorithms to predict the risk of hospital admission, seem to be more focused on substitution of routine follow-up visits [89]. Therefore, future economic evaluation models should probably explicitly consider RPM’s substitution aim.

## Conclusions

This study demonstrates that both from a healthcare and societal perspective, RPM can be cost-effective in the management of HF in the Netherlands, the UK, and Germany even if all costs (medical and non-medical) in life years gained are included.

## Electronic supplementary material

Below is the link to the electronic supplementary material.

Supplementary Material 1

## Data Availability

All model inputs and model outputs are available within the main text or supplementary material. The economic model was developed in RStudio and Microsoft Excel and is available upon request.

## Code Availability

The economic model was developed in RStudio and Microsoft Excel and is available upon request.

## References

[1] Ponikowski, P., Voors, A.A., Anker, S.D., et al.: 2016 ESC guidelines for the diagnosis and treatment of acute and chronic heart failure: The Task Force for the diagnosis and treatment of acute and chronic heart failure of the European Society of Cardiology (ESC)developed with the special contribution of the Heart Failure Association (HFA) of the ESC. Eur. Heart J. **37**, 2129–2200 (2016) 27206819 10.1093/eurheartj/ehw128

[2] Cijfers hart- en vaatziekten: https://www.hartstichting.nl/hart-en-vaatziekten/feiten-en-cijfers-hart-en-vaatziekten

[3] Lesyuk, W., Kriza, C., Kolominsky-Rabas, P.: Cost-of-illness studies in heart failure: A systematic review 2004–2016. BMC Cardiovasc. Disord. **18**, 74 (2018). 10.1186/s12872-018-0815-3 29716540 10.1186/s12872-018-0815-3 PMC5930493

[4] Cost of Illness: (2017). https://www.volksgezondheidenzorg.info/onderwerp/hartfalen/kosten/zorguitgaven#bronverantwoording

[5] Cowie, M.R.: The heart failure epidemic: A UK perspective. Echo Res. Pract. **4**, R15–20 (2017) 28196811 10.1530/ERP-16-0043 PMC5435875

[6] Störk, S., Handrock, R., Jacob, J., et al.: Epidemiology of heart failure in Germany: A retrospective database study. Clin. Res. Cardiol. **106**, 913–922 (2017) 28748265 10.1007/s00392-017-1137-7 PMC5655572

[7] Clark, R.A., Inglis, S.C., McAlister, F.A., et al.: Telemonitoring or structured telephone support programmes for patients with chronic heart failure: Systematic review and meta-analysis. BMJ. **334**, 942 (2007) 17426062 10.1136/bmj.39156.536968.55 PMC1865411

[8] Bertagnin, E., Greco, A., Bottaro, G., et al.: Remote monitoring for heart failure management during COVID-19 pandemic. IJC Heart Vasculature. **32**, 100724 (2021) 33532544 10.1016/j.ijcha.2021.100724 PMC7843025

[9] Mohebali, D., Kittleson, M.M.: Remote monitoring in heart failure: Current and emerging technologies in the context of the pandemic. Heart. **107**, 366–372 (2021) 33431425 10.1136/heartjnl-2020-318062

[10] Celler, B.G., Sparks, R.S.: Home Telemonitoring of Vital signs—Technical challenges and future directions. IEEE J. Biomed. Health Inf. **19**, 82–91 (2015) 10.1109/JBHI.2014.2351413 25163076

[11] Kitsiou, S., Paré, G., Jaana, M.: Effects of Home Telemonitoring interventions on patients with Chronic Heart failure: An overview of systematic reviews. J. Med. Internet Res. **17**, e63 (2015) 25768664 10.2196/jmir.4174 PMC4376138

[12] Grustam, A.S., Severens, J.L., De Massari, D., et al.: Cost-effectiveness analysis in Telehealth: A comparison between Home Telemonitoring, Nurse Telephone Support, and Usual Care in Chronic Heart failure management. Value Health. **21**, 772–782 (2018) 30005749 10.1016/j.jval.2017.11.011

[13] Albuquerque de Almeida, F., Corro Ramos, I., Rutten-van Mölken, M., et al.: Modeling early warning systems: Construction and validation of a Discrete Event Simulation Model for Heart failure. Value Health. **24**, 1435–1445 (2021) 34593166 10.1016/j.jval.2021.04.004

[14] Thokala, P., Baalbaki, H., Brennan, A., et al.: Telemonitoring after discharge from hospital with heart failure: Cost-effectiveness modelling of alternative service designs. BMJ Open. **3**, e003250 (2013) 24048626 10.1136/bmjopen-2013-003250 PMC3780300

[15] Koehler, F., Koehler, K., Prescher, S., et al.: Mortality and morbidity 1 year after stopping a remote patient management intervention: Extended follow-up results from the telemedical interventional management in patients with heart failure II (TIM-HF2) randomised trial. Lancet Digit. Health. **2**, e16–24 (2020) 33328035 10.1016/S2589-7500(19)30195-5

[16] Cowie, M.R., Simon, M., Klein, L., et al.: The cost-effectiveness of real-time pulmonary artery pressure monitoring in heart failure patients: A European perspective. Eur. J. Heart Fail. **19**, 661–669 (2017) 28176424 10.1002/ejhf.747 PMC5434803

[17] Gingele, A.J., Brunner-la Rocca, H., Ramaekers, B., et al.: Telemonitoring in patients with heart failure: Is there a long-term effect? J. Telemed Telecare. **25**, 158–166 (2019) 29251245 10.1177/1357633X17747641

[18] Boyne, J.J., Di Van Asselt, A., Gorgels, A.P., et al.: Cost–effectiveness analysis of telemonitoring versus usual care in patients with heart failure: The TEHAF–study. J. Telemed Telecare. **19**, 242–248 (2013) 24163233 10.1177/1357633X13495478

[19] Jiang, X., Yao, J., You, J.H.: Telemonitoring Versus Usual Care for Elderly patients with heart failure discharged from the hospital in the United States: Cost-effectiveness analysis. JMIR Mhealth Uhealth. **8**, e17846 (2020) 32407288 10.2196/17846 PMC7381019

[20] Jiang, X., Yao, J., You, J.H.S.: Cost-effectiveness of a Telemonitoring Program for patients with heart failure during the COVID-19 pandemic in Hong Kong: Model Development and Data Analysis. J. Med. Internet Res. **23**, e26516 (2021) 33656440 10.2196/26516 PMC7931824

[21] Henderson, C., Knapp, M., Fernandez, J.-L., et al.: Cost effectiveness of telehealth for patients with long term conditions (whole systems Demonstrator telehealth questionnaire study): Nested economic evaluation in a pragmatic, cluster randomised controlled trial. BMJ. **346**, f1035–f1035 (2013) 23520339 10.1136/bmj.f1035

[22] Cleland, J.G.F., Louis, A.A., Rigby, A.S., et al.: Noninvasive Home Telemonitoring for patients with heart failure at high risk of recurrent admission and death. J. Am. Coll. Cardiol. **45**, 1654–1664 (2005) 15893183 10.1016/j.jacc.2005.01.050

[23] Klersy, C., De Silvestri, A., Gabutti, G., et al.: Economic impact of remote patient monitoring: An integrated economic model derived from a meta-analysis of randomized controlled trials in heart failure. Eur. J. Heart Fail. **13**, 450–459 (2011) 21193439 10.1093/eurjhf/hfq232

[24] Goehler, A., Geisler, B.P., Manne, J.M., et al.: Decision-Analytic models to simulate health outcomes and costs in Heart failure: A systematic review. PharmacoEconomics. **29**, 753–769 (2011) 21557632 10.2165/11585990-000000000-00000

[25] Jönsson, B.: Ten arguments for a societal perspective in the economic evaluation of medical innovations. Eur. J. Health Econ. **10**, 357–359 (2009) 19618224 10.1007/s10198-009-0173-2

[26] Hakkaart-van Roijen, L., Van der Linden, N., Bouwmans, C., et al.: Kostenhandleiding: Methodologie Van kostenonderzoek en referentieprijzen voor economische evaluaties in de gezondheidszorg. Zorginstituut Nederland (2016)

[27] de Vries, L.M., van Baal, P.H.M., Brouwer, W.B.F.: Future costs in cost-effectiveness analyses: Past, Present. Future PharmacoEconomics. **37**, 119–130 (2019) 30474803 10.1007/s40273-018-0749-8 PMC6386050

[28] Meltzer, D.: Accounting for future costs in medical cost-effectiveness analysis. J. Health. Econ. **16**, 33–64 (1997) 10167344 10.1016/s0167-6296(96)00507-3

[29] Garber, A.M., Phelps, C.E.: Economic foundations of cost-effectiveness analysis. J. Health Econ. **16**, 1–31 (1997) 10167341 10.1016/s0167-6296(96)00506-1

[30] Nyman, J.A.: Should the consumption of survivors be included as a cost in cost-utility analysis? Health Econ. **13**, 417–427 (2004) 15127422 10.1002/hec.850

[31] van Baal, P., Meltzer, D., Brouwer, W.: Future costs, Fixed Healthcare Budgets, and the decision rules of cost-effectiveness analysis. Health Econ. **25**, 237–248 (2016) 25533778 10.1002/hec.3138

[32] Lee, R.H.: Future costs in cost effectiveness analysis. J. Health Econ. **27**, 809–818 (2008) 18201785 10.1016/j.jhealeco.2007.09.011

[33] Nyman, J.A.: Measurement of QALYS and the welfare implications of survivor consumption and leisure forgone. Health Econ. **20**, 56–67 (2011) 19946890 10.1002/hec.1567

[34] Gandjour, A., Lauterbach, K.W.: Does prevention save costs? Considering deferral of the expensive last year of life. J. Health. Econ. **24**, 715–724 (2005) 15960993 10.1016/j.jhealeco.2004.11.009

[35] Jiao, B., Basu, A.: Catalog of age- and medical Condition—Specific Healthcare costs in the United States to inform future costs calculations in cost-effectiveness analysis. Value Health. **24**, 957–965 (2021) 34243839 10.1016/j.jval.2021.03.006

[36] Kellerborg, K., Perry-Duxbury, M., de Vries, L., et al.: Practical Guidance for including future costs in economic evaluations in the Netherlands: Introducing and applying PAID 3.0. Value Health: J. Int. Soc. Pharmacoeconomics Outcomes Res. **23**, 1453–1461 (2020) 10.1016/j.jval.2020.07.004 33127016

[37] Perry-Duxbury, M., Asaria, M., Lomas, J., et al.: Cured today, Ill tomorrow: A method for including future unrelated medical costs in economic evaluation in England and Wales. Value Health. **23**, 1027–1033 (2020) 32828214 10.1016/j.jval.2020.05.006

[38] Blakely, T., Atkinson, J., Kvizhinadze, G., et al.: Updated New Zealand health system cost estimates from health events by sex, age and proximity to death: Further improvements in the age of ‘big data’. N Z. Med. J. **128**, 13–23 (2015) 26411843

[39] Tew, M., Clarke, P., Thursky, K., et al.: Incorporating Future Medical costs: Impact on cost-effectiveness analysis in Cancer patients. PharmacoEconomics. **37**, 931–941 (2019) 30864067 10.1007/s40273-019-00790-9

[40] Rappange, D.R., van Baal, P.H.M., van Exel, N.J.A., et al.: Unrelated medical costs in Life-Years gained: Should they be included in economic evaluations of Healthcare interventions? PharmacoEconomics. **26**, 815–830 (2008) 18793030 10.2165/00019053-200826100-00003

[41] Briggs, A.D.M., Scarborough, P., Wolstenholme, J.: Estimating comparable English healthcare costs for multiple diseases and unrelated future costs for use in health and public health economic modelling. PLoS ONE. **13**, e0197257 (2018) 29795586 10.1371/journal.pone.0197257 PMC5967835

[42] Olchanski, N., Zhong, Y., Cohen, J.T., et al.: The peculiar economics of life-extending therapies: A review of costing methods in health economic evaluations in oncology. Expert Rev. PharmacoEcon. Outcomes Res. **15**, 931–940 (2015) 26478989 10.1586/14737167.2015.1102633

[43] Manns, B., Meltzer, D., Taub, K., et al.: Illustrating the impact of including future costs in economic evaluations: An application to end-stage renal disease care. Health Econ. **12**, 949–958 (2003) 14601157 10.1002/hec.790

[44] Ramos, I.C., Versteegh, M.M., de Boer, R.A., et al.: Cost effectiveness of the angiotensin receptor neprilysin inhibitor Sacubitril/Valsartan for patients with chronic heart failure and reduced ejection fraction in the Netherlands: A Country Adaptation Analysis under the former and current Dutch pharmacoeconomic guidelines. Value Health. **20**, 1260–1269 (2017) 29241885 10.1016/j.jval.2017.05.013

[45] Grima, D.T., Bernard, L.M., Dunn, E.S., et al.: Cost-effectiveness analysis of therapies for chronic kidney disease patients on Dialysis: A case for excluding Dialysis costs. PharmacoEconomics. **30**, 981–989 (2012) 22946789 10.2165/11599390-000000000-00000

[46] Kruse, M., Sørensen, J., Gyrd-Hansen, D.: Future costs in cost-effectiveness analysis: An empirical assessment. Eur. J. Health Econ. **13**, 63–70 (2012) 20878202 10.1007/s10198-010-0280-0 PMC3249583

[47] van Baal, P.H.M., Hoogendoorn, M., Fischer, A.: Preventing dementia by promoting physical activity and the long-term impact on health and social care expenditures. Prev. Med. **85**, 78–83 (2016) 26825761 10.1016/j.ypmed.2016.01.013

[48] Jiang, S., Wang, Y., Zhou, J., et al.: Incorporating future unrelated medical costs in cost-effectiveness analysis in China. BMJ Glob Health. **6**, e006655 (2021) 34702751 10.1136/bmjgh-2021-006655 PMC8549663

[49] Inglis, S.C., Clark, R.A., Dierckx, R., et al.: Structured telephone support or non-invasive telemonitoring for patients with heart failure. Cochrane Database Syst. Reviews. **2015** (2015). 10.1002/14651858.CD007228.pub3 10.1002/14651858.CD007228.pub3 PMC8482064 26517969

[50] Scholte, N.T.B., Gürgöze, M.T., Aydin, D., et al.: Telemonitoring for heart failure: A meta-analysis. Eur. Heart J. **44**, 2911–2926 (2023) 37216272 10.1093/eurheartj/ehad280 PMC10424885

[51] Brons, M., Ten Klooster, I., van Gemert-Pijnen, L., et al.: Patterns in the Use of Heart failure telemonitoring: Post Hoc Analysis of the e-Vita Heart failure trial. JMIR Cardio. **7**, e41248 (2023) 36719715 10.2196/41248 PMC9929726

[52] Kansal, A.R., Cowie, M.R., Kielhorn, A., et al.: Cost-effectiveness of Ivabradine for Heart failure in the United States. JAHA. **5**, e003221 (2016) 27153871 10.1161/JAHA.116.003221 PMC4889192

[53] Di Tanna, G.L., Urbich, M., Wirtz, H.S., et al.: Health State Utilities of patients with Heart failure: A systematic literature review. PharmacoEconomics. **39**, 211–229 (2021) 33251572 10.1007/s40273-020-00984-6 PMC7867520

[54] International Monetary Fund: Inflation rate, average consumer prices (Annual percent change)

[55] Zhao, Y., Feng, H., Qu, J., et al.: A systematic review of pharmacoeconomic guidelines. J. Med. Econ. **21**, 85–96 (2018) 28959910 10.1080/13696998.2017.1387118

[56] Zorginstituut Nederland: Richtlijn voor het uitvoeren van economische evaluaties in de gezondheidszorg: (2016)

[57] van Baal, P.H.M., Wong, A., Slobbe, L.C.J., et al.: Standardizing the inclusion of Indirect Medical costs in economic evaluations. PharmacoEconomics. **29**, 175–187 (2011) 21184618 10.2165/11586130-000000000-00000

[58] Mokri, H., Kvamme, I., de Vries, L., et al.: Future medical and non-medical costs and their impact on the cost-effectiveness of life-prolonging interventions: A comparison of five European countries. Eur. J. Health Econ. Published Online First: 4 August. (2022). 10.1007/s10198-022-01501-6 10.1007/s10198-022-01501-6 PMC10198844 35925501

[59] Irene Santi, S., de Groot, P., Bakx: Bram Wouterse, Pieter Van Baal. Informal care Costs According to age and Proximity to Death to Support cost-effectiveness Analyses. Work in progress 10.1007/s40273-022-01233-8 PMC10450016 36725787

[60] Börsch-Supan, A., Brandt, M., Hunkler, C., et al.: Data Resource Profile: The Survey of Health, Ageing and Retirement in Europe (SHARE). Int. J. Epidemiol. **42**, 992–1001 (2013) 23778574 10.1093/ije/dyt088 PMC3780997

[61] Wong, A., van Baal, P.H.M., Boshuizen, H.C., et al.: Exploring the influence of proximity to death on disease-specific hospital expenditures: A carpaccio of red herrings. Health Econ. **20**, 379–400 (2011) 20232289 10.1002/hec.1597

[62] Husereau, D., Drummond, M., Augustovski, F., et al.: Consolidated Health Economic evaluation reporting standards 2022 (CHEERS 2022) statement: Updated reporting guidance for health economic evaluations. BMC Med. **20**, 23 (2022) 35022047 10.1186/s12916-021-02204-0 PMC8753858

[63] Wouterse, B., van Baal, P., Versteegh, M., et al.: The Value of Health in a cost-effectiveness analysis: Theory Versus Practice. Pharmacoeconomics. **41**, 607–617 (2023) 37072598 10.1007/s40273-023-01265-8 PMC10163089

[64] Woods, B., Revill, P., Sculpher, M., et al.: Country-Level cost-effectiveness thresholds: Initial estimates and the need for further research. Value Health. **19**, 929–935 (2016) 27987642 10.1016/j.jval.2016.02.017 PMC5193154

[65] Claxton, K., Martin, S., Soares, M., et al.: Methods for the estimation of the National Institute for Health and Care Excellence cost-effectiveness threshold. Health Technol. Assess. **19**, 1–504 (2015) 25692211 10.3310/hta19140 PMC4781395

[66] Thokala, P., Ochalek, J., Leech, A.A., et al.: Cost-effectiveness thresholds: The past, the Present and the future. Pharmacoeconomics. **36**, 509–522 (2018) 29427072 10.1007/s40273-017-0606-1

[67] Zorginstituut Nederland: Ziektelast in de praktijk. De theorie en praktijk van het berekenen van ziektelast bij pakketbeoordelingen. https://www.zorginstituutnederland.nl/binaries/zinl/documenten/rapport/2018/05/07/ziektelast-in-de-praktijk/Ziektelast+in+de+praktijk_definitief.pdf

[68] National Institute for Health and Care Excellence. Guide to the Methods of Technology Appraisal: (2013). https://www.nice.org.uk/process/pmg9/chapter/foreword 27905712

[69] NICE health technology evaluations: the manual: (2022). https://www.nice.org.uk/process/pmg36/chapter/introduction-to-health-technology-evaluation

[70] van Baal, P., Perry-Duxbury, M., Bakx, P., et al.: A cost-effectiveness threshold based on the marginal returns of cardiovascular hospital spending. Health Econ. **28**, 87–100 (2019) 30273967 10.1002/hec.3831 PMC6585934

[71] Pichon-Riviere, A., Drummond, M., Palacios, A., et al.: Determining the efficiency path to universal health coverage: Cost-effectiveness thresholds for 174 countries based on growth in life expectancy and health expenditures. Lancet Glob Health. **11**, e833–e842 (2023) 37202020 10.1016/S2214-109X(23)00162-6

[72] McMurray, J.J.V., Trueman, D., Hancock, E., et al.: Cost-effectiveness of sacubitril/valsartan in the treatment of heart failure with reduced ejection fraction. Heart. **104**, 1006–1013 (2018) 29269379 10.1136/heartjnl-2016-310661 PMC5992367

[73] Peters-Klimm, F., Halmer, A., Flessa, S., et al.: What drives the costs of heart failure care in Germany? A health services cost analysis. J. Public. Health. **20**, 653–660 (2012)

[74] Wilson, E., Thalanany, M., Shepstone, L., et al.: Befriending carers of people with dementia: A cost utility analysis. Int. J. Geriat Psychiatry. **24**, 610–623 (2009) 10.1002/gps.2164 19101921

[75] Kellerborg, K., Wouterse, B., Brouwer, W., et al.: Estimating the costs of non-medical consumption in life-years gained for economic evaluations. Soc. Sci. Med. **289**, 114414 (2021) 34563871 10.1016/j.socscimed.2021.114414

[76] McNamara, S., Schneider, P.P., Love-Koh, J., et al.: Quality-adjusted life expectancy norms for the English Population. Value Health ;**S1098301522021015**. (2022) 10.1016/j.jval.2022.07.005 35965226

[77] Meltzer, D.O., Smith, P.C.: Theoretical issues relevant to the Economic Evaluation of Health Technologies. Handb. Health Econ. Elsevier **2011**:433–469. https://ideas.repec.org/h/eee/heachp/2-433.html

[78] Claxton, K., Palmer, S., Sculpher, M., Walker, S.: Appropriate perspectives for health care decisions. (2010). http://www.york.ac.uk/inst/che/pdf/rp54.pdf

[79] Ryen, L., Svensson, M.: The willingness to pay for a quality adjusted Life Year: A review of the empirical literature. Health Econ. **24**, 1289–1301 (2015) 25070495 10.1002/hec.3085

[80] Vallejo-Torres, L., García-Lorenzo, B., Castilla, I., et al.: On the estimation of the cost-effectiveness threshold: Why, what, how? Value Health. **19**, 558–566 (2016) 27565273 10.1016/j.jval.2016.02.020

[81] Jessica Ochalek, J., Lomas, K., Claxton: Estimating health opportunity costs in low-income and middle-income countries: A novel approach and evidence from cross-country data. BMJ Global Health. **3**, e000964 (2018) 30483412 10.1136/bmjgh-2018-000964 PMC6231096

[82] McDonagh, T.A., Metra, M., Adamo, M., et al.: 2021 ESC guidelines for the diagnosis and treatment of acute and chronic heart failure. Eur. Heart J. **42**, 3599–3726 (2021) 34447992 10.1093/eurheartj/ehab368

[83] Steiner, B., Neumann, A., Pelz, Y., et al.: Challenges in heart failure care in four European countries: A comparative study. Eur. J. Pub. Health. **33**, 448–454 (2023) 37164632 10.1093/eurpub/ckad059 PMC10234648

[84] Papanicolas, I., Mossialos, E., Gundersen, A., et al.: Performance of UK National Health Service compared with other high income countries: Observational study. BMJ ;l6326. (2019) 10.1136/bmj.l6326 PMC6880250 31776110

[85] Louis, A.A., Turner, T., Gretton, M., et al.: A systematic review of telemonitoring for the management of heart failure. Eur. J. Heart Fail. **5**, 583–590 (2003) 14607195 10.1016/s1388-9842(03)00160-0

[86] Zhao, Q., Chen, C., Zhang, J., et al.: Effects of self-management interventions on heart failure: Systematic review and meta-analysis of randomized controlled trials – reprint. Int. J. Nurs. Stud. **116**, 103909 (2021) 33642066 10.1016/j.ijnurstu.2021.103909

[87] Brahmbhatt, D.H., Cowie, M.R.: Remote management of Heart failure: An overview of Telemonitoring technologies. Card Fail. Rev. **5**, 86–92 (2019) 31179018 10.15420/cfr.2019.5.3 PMC6545972

[88] Auener, S.L., Remers, T.E.P., van Dulmen, S.A., et al.: The Effect of Noninvasive Telemonitoring for Chronic Heart failure on Health Care utilization: Systematic review. J. Med. Internet Res. **23**, e26744 (2021) 34586072 10.2196/26744 PMC8515232

[89] Albuquerque de Almeida, F., Corro Ramos, I., Al, M., et al.: Home Telemonitoring and a diagnostic algorithm in the management of Heart failure in the Netherlands: Cost-effectiveness analysis. JMIR Cardio. **6**, e31302 (2022) 35925670 10.2196/31302 PMC9389378

