# Supplementary material for: The impact of different perspectives on the cost-effectiveness of remote patient monitoring for patients with heart failure in different European countries
Source: Eur J Health Econ. 2024 May 3;26(1):71–85. doi: 10.1007/s10198-024-01690-2 (PMC11743354; doi:10.1007/s10198-024-01690-2)
Supplement: Supplementary file 1 — Supplementary Material 1 [file 10198_2024_1690_MOESM1_ESM.docx]

**Electronic Supplementary Information (SI)**

Manuscript title: The impact of different perspectives on the cost-effectiveness of remote patient monitoring for patients with heart failure in different European countries.

Hamraz Mokri^a^, Pieter van Baal^a^, Maureen Rutten-van Mölken^a,b^

a – Erasmus School of Health policy and Management (ESHPM), Erasmus University Rotterdam, Rotterdam, The Netherlands

b – Institute for Medical Technology Assessment(iMTA), Erasmus University Rotterdam, Rotterdam, The Netherlands

Corresponding author: Mokri@eshpm.eur.nl

**Supplementary table 1*.*** Input costs by age and per country

|  |  | Netherlands |  |  | UK |  |  | Germany |  |
| --- | --- | --- | --- | --- | --- | --- | --- | --- | --- |
| Age | Informal care | Unrelated medical | Non-medical | Informal care | Unrelated medical | Non-medical | Informal care | Unrelated medical | Non-medical |
| 70 | € 1,698 | € 5,693 | € 10,179 | € 1,635 | € 4,008 | € 14,907 | € 2,868 | € 3,151 | € 12,827 |
| 71 | € 3,300 | € 6,001 | € 10,150 | € 3,178 | € 4,081 | € 14,915 | € 5,574 | € 3,107 | € 12,657 |
| 72 | € 4,815 | € 6,397 | € 10,124 | € 4,636 | € 4,378 | € 14,930 | € 8,132 | € 3,379 | € 12,452 |
| 73 | € 6,249 | € 6,885 | € 10,100 | € 6,018 | € 4,859 | € 14,951 | € 10,554 | € 3,576 | € 12,214 |
| 74 | € 7,610 | € 7,451 | € 10,079 | € 7,328 | € 5,427 | € 14,978 | € 12,853 | € 3,681 | € 11,950 |
| 75 | € 8,905 | € 8,037 | € 10,059 | € 8,575 | € 6,013 | € 15,009 | € 15,040 | € 3,749 | € 11,663 |
| 76 | € 10,139 | € 8,602 | € 10,041 | € 9,764 | € 6,571 | € 15,042 | € 17,124 | € 4,007 | € 11,360 |
| 77 | € 11,319 | € 9,146 | € 10,025 | € 10,900 | € 7,106 | € 15,075 | € 19,117 | € 3,944 | € 11,043 |
| 78 | € 12,449 | € 9,745 | € 10,009 | € 11,988 | € 7,622 | € 15,108 | € 21,026 | € 4,141 | € 10,717 |
| 79 | € 13,535 | € 10,516 | € 9,995 | € 13,033 | € 8,146 | € 15,137 | € 22,859 | € 4,239 | € 10,387 |
| 80 | € 14,580 | € 11,460 | € 9,981 | € 14,040 | € 8,661 | € 15,137 | € 24,623 | € 4,384 | € 10,387 |
| 81 | € 15,588 | € 12,634 | € 9,968 | € 15,011 | € 9,183 | € 15,137 | € 26,326 | € 4,535 | € 10,387 |
| 82 | € 16,563 | € 13,988 | € 9,955 | € 15,950 | € 9,659 | € 15,137 | € 27,973 | € 4,614 | € 10,387 |
| 83 | € 17,509 | € 15,406 | € 9,943 | € 16,860 | € 10,111 | € 15,137 | € 29,570 | € 4,721 | € 10,387 |
| 84 | € 18,427 | € 16,905 | € 9,929 | € 17,745 | € 10,716 | € 15,137 | € 31,122 | € 5,214 | € 10,387 |
| 85 | € 19,322 | € 18,582 | € 9,912 | € 18,606 | € 11,558 | € 15,137 | € 32,633 | € 5,174 | € 10,387 |
| 86 | € 20,194 | € 20,222 | € 9,892 | € 19,446 | € 12,404 | € 15,137 | € 34,106 | € 5,056 | € 10,387 |
| 87 | € 21,047 | € 21,861 | € 9,868 | € 20,267 | € 13,158 | € 15,137 | € 35,546 | € 4,878 | € 10,387 |
| 88 | € 21,881 | € 23,643 | € 9,842 | € 21,071 | € 13,788 | € 15,137 | € 36,954 | € 4,816 | € 10,387 |
| 89 | € 22,698 | € 25,599 | € 9,812 | € 21,858 | € 14,266 | € 15,137 | € 38,335 | € 4,748 | € 10,387 |
| 90 | € 23,500 | € 27,698 | € 9,779 | € 22,630 | € 14,715 | € 15,137 | € 39,689 | € 4,702 | € 10,387 |
| 91 | € 24,287 | € 29,921 | € 9,742 | € 23,388 | € 15,233 | € 15,137 | € 41,019 | € 4,654 | € 10,387 |
| 92 | € 25,061 | € 32,218 | € 9,702 | € 24,133 | € 15,912 | € 15,137 | € 42,325 | € 4,598 | € 10,387 |
| 93 | € 25,821 | € 34,487 | € 9,659 | € 24,865 | € 16,709 | € 15,137 | € 43,609 | € 4,537 | € 10,387 |
| 94 | € 26,569 | € 36,782 | € 9,612 | € 25,585 | € 17,384 | € 15,137 | € 44,872 | € 4,471 | € 10,387 |
| 95 | € 27,304 | € 39,255 | € 9,562 | € 26,293 | € 17,865 | € 15,137 | € 46,114 | € 4,401 | € 10,387 |

| 96 | € 28,027 | € 41,701 | € 9,510 | € 26,989 | € 17,995 | € 15,137 | € 47,335 | € 4,326 | € 10,387 |
| --- | --- | --- | --- | --- | --- | --- | --- | --- | --- |
| 97 | € 28,738 | € 39,334 | € 9,454 | € 27,673 | € 17,603 | € 15,137 | € 48,535 | € 4,243 | € 10,387 |
| 98 | € 29,436 | € 39,827 | € 9,396 | € 28,346 | € 17,506 | € 15,137 | € 49,714 | € 4,150 | € 10,387 |
| 99 | € 30,121 | € 41,010 | € 9,335 | € 29,006 | € 18,348 | € 15,137 | € 50,871 | € 4,273 | € 10,387 |
| 100 | € 30,121 | € 41,010 | € 9,335 | € 29,006 | € 18,348 | € 15,137 | € 50,871 | € 4,273 | € 10,387 |

|  | Base-case | Lower bound | Upper bound | Assumptions and references |
| --- | --- | --- | --- | --- |
| Risk ratio all-cause mortality | 0.80 | 0.94 | 0.68 | 95% confidence interval is used. [1] |
| Risk ratio HF-related hospitalization | 0.71 | 0.60 | 0.83 | 95% confidence interval is used. [1] |
| Utility values | 0.6970 | 0.5576 | 0.8364 | 20% increase for the upper bound and 20% decrease for the lower bound. [2] |
| HF-related hospitalization costs |  |  |  | 20% increase for the upper bound and 20% decrease for the lower bound. |
| -The Netherlands  -UK  -Germany | €4,937  €2,511  €4,635 | €3,950  €2,009  €3,708 | €5,924  €3,013  €5,562 | [3]  [4]  [4] |
| HF-related non-hospitalization costs: |  |  |  | 20% increase for the upper bound and 20% decrease for the lower bound. |
| -The Netherlands  -UK  -Germany | €378  €565  €393 | €302  €452  €314 | €454  €678  €472 | [5,6]  [7]  [6] |
| Intervention costs | €417.37 | €334 | €501 | 20% increase for the upper bound and 20% decrease for the lower bound. [2] |
| Informal care costs | Age-specific | Age-specific | Age-specific | 20% increase for the upper bound and 20% decrease for the lower bound [8,9] |
| Costs of non-medical consumption | Age-specific | Age-specific | Age-specific | 20% increase for the upper bound and 20% decrease for the lower bound [10–12] |
| Other medical costs | Age-specific | Age-specific | Age-specific | 20% increase for the upper bound and 20% decrease for the lower bound [10–12] |

**Supplementary table 2**. Inputs for the one-way sensitivity analysis for the Netherlands, the UK and Germany

**Supplementary Fig 1** Tornado diagram for the one-way sensitivity analysis showing the variation in the base-case cost-effectiveness results for (a) the Netherlands, (b) The UK, (c) Germany, from a societal perspective.

A larger bar indicates a greater impact on the ICER. The dotted line indicates the base-case ICER.

**Reference:**

1 Inglis SC, Clark RA, Dierckx R, *et al.* Structured telephone support or non-invasive telemonitoring for patients with heart failure. *Cochrane Database of Systematic Reviews* 2015;**2015**. doi:10.1002/14651858.CD007228.pub3

2 Grustam AS, Severens JL, De Massari D, *et al.* Cost-Effectiveness Analysis in Telehealth: A Comparison between Home Telemonitoring, Nurse Telephone Support, and Usual Care in Chronic Heart Failure Management. *Value in Health* 2018;**21**:772–82. doi:10.1016/j.jval.2017.11.011

3 Albuquerque de Almeida F, Corro Ramos I, Rutten-van Mölken M, *et al.* Modeling Early Warning Systems: Construction and Validation of a Discrete Event Simulation Model for Heart Failure. *Value in Health* 2021;**24**:1435–45. doi:10.1016/j.jval.2021.04.004

4 Cowie MR, Simon M, Klein L, *et al.* The cost-effectiveness of real-time pulmonary artery pressure monitoring in heart failure patients: a European perspective. *Eur J Heart Fail* 2017;**19**:661–9. doi:10.1002/ejhf.747

5 Ramos IC, Versteegh MM, de Boer RA, *et al.* Cost Effectiveness of the Angiotensin Receptor Neprilysin Inhibitor Sacubitril/Valsartan for Patients with Chronic Heart Failure and Reduced Ejection Fraction in the Netherlands: A Country Adaptation Analysis Under the Former and Current Dutch Pharmacoeconomic Guidelines. *Value in Health* 2017;**20**:1260–9. doi:10.1016/j.jval.2017.05.013

6 McMurray JJV, Trueman D, Hancock E, *et al.* Cost-effectiveness of sacubitril/valsartan in the treatment of heart failure with reduced ejection fraction. *Heart* 2018;**104**:1006–13. doi:10.1136/heartjnl-2016-310661

7 Peters-Klimm F, Halmer A, Flessa S, *et al.* What drives the costs of heart failure care in Germany? A health services cost analysis. *J Public Health* 2012;**20**:653–60. doi:10.1007/s10389-012-0501-3

8 Irene Santi, Saskia de Groot, Pieter Bakx, Bram Wouterse, Pieter van Baal. Informal care costs according to age and proximity to death to support cost-effectiveness analyses. Work in progress.

9 Wilson E, Thalanany M, Shepstone L, *et al.* Befriending carers of people with dementia: a cost utility analysis. *Int J Geriat Psychiatry* 2009;**24**:610–23. doi:10.1002/gps.2164

10 Kellerborg K, Perry-Duxbury M, Vries L de, *et al.* Practical Guidance for Including Future Costs in Economic Evaluations in The Netherlands: Introducing and Applying PAID 3.0. *Value in health : the journal of the International Society for Pharmacoeconomics and Outcomes Research* 2020;**23**:1453–61. doi:S1098-3015(20)32212-9 [pii]

11 Mokri H, Kvamme I, de Vries L, *et al.* Future medical and non-medical costs and their impact on the cost-effectiveness of life-prolonging interventions: a comparison of five European countries. *Eur J Health Econ* Published Online First: 4 August 2022. doi:10.1007/s10198-022-01501-6

12 van Baal PHM, Wong A, Slobbe LCJ, *et al.* Standardizing the Inclusion of Indirect Medical Costs in Economic Evaluations. *PharmacoEconomics* 2011;**29**:175–87. doi:10.2165/11586130-000000000-00000
